# Pan-chloroplast genomes of *Hordeum* reveal insights into chloroplast evolution in the Poaceae family

**DOI:** 10.3389/fpls.2026.1881945

**Published:** 2026-07-20

**Authors:** Qingwei Du, Zhengjiao Zhang, Ruifen Li

**Affiliations:** 1 Beijing Key Laboratory of Agricultural Genetic Resources and Biotechnology, Beijing Key Laboratory of Crop Molecular Design and Intelligent Breeding, Institute of Biotechnology, Beijing Academy of Agriculture and Forestry Sciences, Beijing, China; 2 College of Agriculture, Yangtze University, Jingzhou, China

**Keywords:** *Hordeum*, nucleotide diversity, pan–chloroplast genome, repeat sequences, selective pressure, structural variation

## Abstract

Chloroplast genomes are widely used in phylogenetic and evolutionary studies due to their conserved structure and maternal inheritance. However, a comprehensive pan-chloroplast analysis across the genus *Hordeum* remains lacking. In this study, we assembled and annotated 43 chloroplast genomes representing 12 *Hordeum* species, together with 18 chloroplast genomes from other Poaceae species, to investigate structural variation, repeat sequences, codon usage, RNA editing, selective pressure, and nucleotide diversity. All chloroplast genomes exhibited the typical quadripartite structure with genome sizes ranging from 136,461 bp to 137,149 bp in *Hordeum*. The lengths of LSC and IR regions showed strong positive correlations with chloroplast genome size (r = 0.853 and 0.857, respectively). IR boundaries were highly conserved within *Hordeum*, whereas three distinct boundary types were identified across Poaceae. Pentanucleotide simple sequence repeats (SSRs) were universally present in *Hordeum* but absent in some other Poaceae species, representing a candidate *Hordeum*-enriched marker pending broader validation. Based on the newly assembled chloroplast genomes of *Hordeum*, the phylogenetic relationships were reappraised. A total of 66 protein−coding genes contained RNA editing sites, with *ndhB* harboring the most edits in *Hordeum*. All examined protein–coding genes showed *Ka/Ks* values below 1, while ribosomal subunit genes exhibited the highest interspecific variation in *Ka/Ks*. *Hordeum bulbosum* displayed the highest intraspecific nucleotide diversity and haplotype diversity, whereas cultivated *Hordeum vulgare* showed extremely low diversity. Several hypervariable regions were identified as candidate DNA barcodes. This pan−chloroplast genome study provides new insights into the structural and evolutionary dynamics of *Hordeum* chloroplast genomes and offers valuable genomic resources for phylogeny, germplasm identification, and adaptive evolution in the genus.

## 1 Introduction

Chloroplasts are semi–autonomous organelles possessing an independent genetic system. Their most fundamental function is to drive photosynthesis, converting solar energy into chemical energy, and they serve as platforms for the synthesis of fatty acids, amino acids, hormones, vitamins, and various secondary metabolites (Daniell et al., 2016). The typical chloroplast genome is a circular molecule with a quadripartite structure, comprising a large single–copy (LSC) region, a small single–copy (SSC) region, and two inverted repeat (IR) regions, usually ranging from 120 to 160 kb in size. Although the overall chloroplast genome structure is highly conserved across land plants, lineage-specific variations such as gene loss, genome rearrangement, IR expansion/contraction, and intron loss have occurred during evolution (Wicke et al., 2011). Compared with the nuclear genome, the chloroplast genome evolves more slowly, is structurally stable, is maternally inherited, and does not undergo recombination. These features make it an ideal molecular marker for phylogenetic reconstruction, species identification, DNA barcode development, and population genetic diversity studies (Xu et al., 2026). The rapid development of high-throughput sequencing technologies has enabled the decoding of thousands of complete chloroplast genomes, laying a solid foundation for understanding their structure, function, and evolutionary patterns.

The grass family (Poaceae) is one of the five most species–rich families of angiosperms, widely adapted to diverse climatic zones across the globe. It includes major cereal crops such as rice, wheat, and maize, as well as many high–quality forages, energy plants, and species used for ecological restoration. Chloroplast genomes of Poaceae are generally conserved, exhibiting the typical quadripartite circular configuration, with sizes ranging from 125 to 165 kb, GC content of approximately 37–40%, and encoding 110–130 genes (Hu et al., 2023). Although numerous studies have focused on the chloroplast genomes of individual grass species, systematic comparisons across different Poaceae lineages remain relatively scarce. Notably, different groups within Poaceae display clear lineage–specific variations in gene composition, IR boundary positions, and repeat distribution (Geng et al., 2026). Systematic analysis of these variations based on pan–chloroplast approaches can not only elucidate the evolutionary drivers of Poaceae chloroplast genomes, but also provide theoretical references for developing lineage–specific molecular markers and overcoming technical bottlenecks in chloroplast transformation of grasses.

The genus *Hordeum* is an important branch of Poaceae, comprising approximately 45 species or subspecies (Brassac and Blattner, 2015; Reinert et al., 2019). This genus contains the globally cultivated barley (*H. vulgare*) as well as numerous wild relatives carrying desirable traits such as disease resistance and salt tolerance, representing valuable germplasm resources for crop improvement (Feng et al., 2025). Previous phylogenetic studies have mainly relied on nuclear genes (Blattner, 2004), and conflicts exist between trees derived from limited nuclear and chloroplast data (Brassac and Blattner, 2015), suggesting that *Hordeum* may have experienced complex reticulate evolutionary events, such as incomplete lineage sorting or chloroplast capture. However, a comprehensive whole-chloroplast genome phylogenetic analysis of *Hordeum* has not yet been fully explored. Although chloroplast genomes of some *Hordeum* species have been reported (Yuan et al., 2023), systematic, large–scale, cross–species pan–chloroplast analyses are still lacking, which limits our comprehensive understanding of the organizational patterns and evolutionary history of chloroplast genomes in this genus.

To address these gaps, we assembled and annotated 43 chloroplast genomes representing 12 *Hordeum* species (covering wild and cultivated, perennial and annual types) and combined them with 18 chloroplast genomes from closely related Poaceae species to perform a comprehensive pan–chloroplast comparative analysis. The specific objectives were to (1) complete chloroplast genome assembly and annotation, revealing genome structure, IR boundary dynamics, distribution of repeat sequences, and gene content variation; (2) construct a phylogeny using complete chloroplast genomes to evaluate chloroplast genome–based relationships within *Hordeum*; (3) analyze codon usage bias, RNA editing sites, and selective pressure on protein–coding genes, identifying genes that have experienced differential purifying selection; and (4) evaluate intraspecific and interspecific nucleotide diversity and haplotype diversity, pinpointing hypervariable regions. Through these analyses, we aim to systematically dissect the evolutionary drivers of *Hordeum* and Poaceae chloroplast genomes, enrich the chloroplast genome database, and provide new insights into the phylogeny, germplasm identification, and adaptive evolution of the genus *Hordeum*.

## 2 Materials and methods

### 2.1 Chloroplast genome assembly and annotation

Raw sequencing data for *Hordeum* species were obtained from our previous studies (Feng et al., 2025) and the NCBI public database (**Supplementary Table 1**), while chloroplast genomes of other Poaceae species were downloaded from NCBI (**Supplementary Table 1**). Raw reads were first filtered using Fastp v0.12.4 (Chen et al., 2018) with default parameters. Using the published cultivated barley chloroplast genome (NC_008590) as a reference, we assembled the chloroplast genomes of 43 *Hordeum* accessions with NOVOPlasty v4.3.5 (Dierckxsens et al., 2017) and GetOrganelle v1.7.7.0 (Jin et al., 2020). First, GetOrganelle was used to assemble a complete chloroplast genome sequence, which was then used as the reference sequence for a second round of assembly using NOVOPlasty. For the very few conflicting assembled sequences produced by the two software tools, we manually corrected them by mapping the raw sequencing reads back to the sequences generated by each software. Circularization was checked using Bandage v0.8.1 (Wick et al., 2015). Gene annotation of the 43 *Hordeum* chloroplast genomes and 18 Poaceae chloroplast genomes was performed using CPGAVAS2 (Shi et al., 2019) and GeSeq (Tillich et al., 2017) with default parameters, followed by manual correction. OGDraw v1.3.1 (https://chlorobox.mpimp-golm.mpg.de/OGDraw.html) (Greiner et al., 2019) was used to visualize chloroplast genome structures.

### 2.2 Chloroplast genome feature analysis

Codon usage bias was analyzed using CodonW v1.4.2 (Chakraborty et al., 2020), and relative synonymous codon usage (RSCU) was calculated. Three classes of repeats were identified. Simple sequence repeats (SSRs) were detected using MISA v2.1 (Beier et al., 2017) with minimum thresholds of 10, 5, 4, 3, 3, and 3 repeat units for mono-, di-, tri-, tetra-, penta-, and hexanucleotide motifs, respectively. Tandem repeats were identified using Tandem Repeats Finder v4.09.1 (TRF) with default parameters: match = 2, mismatch = 7, InDel = 7, minimum alignment score of 50, and maximum period size of 500. Dispersed long repeats were detected using REPuter (https://bibiserv.cebitec.uni-bielefeld.de/reputer) (Kurtz et al., 2001) with the following parameters: minimum repeat length 30 bp, Hamming distance 3, and maximum computed repeats 50. RNA editing sites were predicted using the online Plant RNA Editing-Prediction Tool (http://www.prepact.de/prepact-main.php) with the BLASTX prediction mode under default parameters.

### 2.3 Phylogenetic analysis

Using *Oryza sativa* as an outgroup, a maximum likelihood (ML) phylogenetic tree was constructed based on the 43 *Hordeum* chloroplast genomes and 18 other Poaceae chloroplast genomes. The sequences of the LSC region, SSC region, and one repeat region were concatenated and aligned using MAFFT v7.310 (Katoh and Standley, 2013). After alignment, the highly divergent regions were removed using trimAl v1.5.0 (Capella-Gutiérrez et al., 2009) and by manual curation. A partition file was created to define the LSC, SSC, and IR regions as separate data blocks. The ML tree was constructed using IQ-TREE v2.1.2 (Minh et al., 2020) under the edge−proportional model (-p option) with automatic partition−model selection and merging test (-m MFP+MERGE). Branch support was assessed using 1000 ultrafast bootstrap replicates and 1000 SH−aLRT tests. The tree was visualized using iTOL v7 (Letunic and Bork, 2007).

### 2.4 Chloroplast genome structural analysis

Using the cultivated barley chloroplast genome (NC_008590) as a reference, we compared the 12 *Hordeum* chloroplast genomes using mVISTA (https://genome.lbl.gov/vista/mvista/submit.shtml) (Frazer et al., 2004) in Shuffle-LAGAN mode. Synteny analysis of chloroplast genomes was performed using MAUVE v1.1.158 (Darling et al., 2004). The boundaries of the LSC, SSC, and IR regions were determined based on the annotation results.

### 2.5 Genetic diversity analysis

Multiple sequence alignment of *Hordeum* chloroplast genomes was performed with MAFFT v7.310. The aligned sequences were imported into DnaSP v6.12.03 (Rozas et al., 2017) with a step size of 25 bp and a sliding window of 100 bp to calculate nucleotide diversity (Pi), haplotype diversity (Hd), and genetic differentiation (Fst). The protein and coding sequences of the 76 protein–coding genes were input into ParaAT v2.0 (Zhang et al., 2012) for pairwise alignment of gene pairs. The alignment results were then entered into KaKs_Calculator v2.0 (Wang et al., 2010) to calculate *Ka/Ks* values. The *Ka/Ks* values were visualized using the R package pheatmap v1.0.12.

## 3 Results

### 3.1 Chloroplast genome assembly, annotation, and characterization

**Figure 1:**
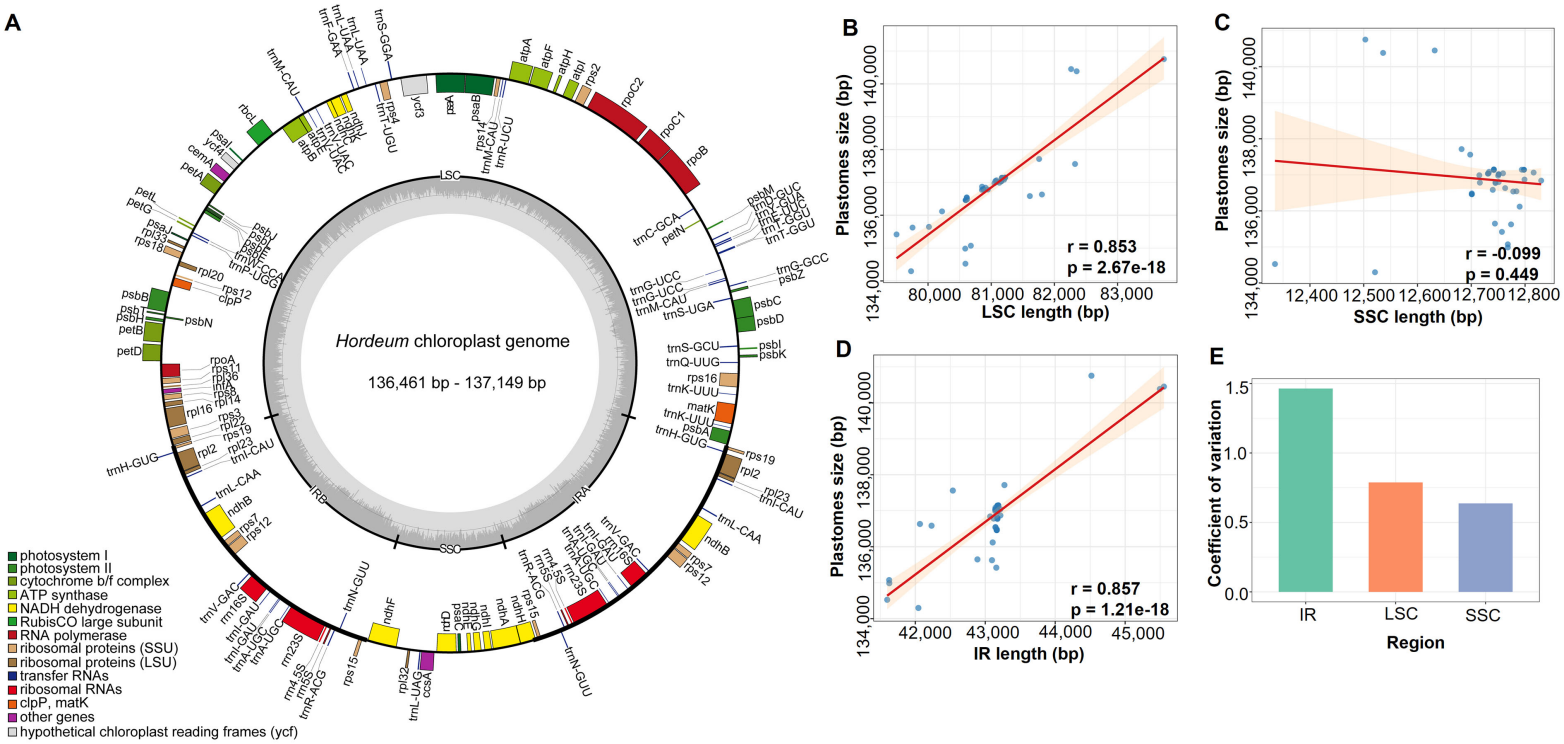
Assembly, annotation, and correlation analysis of chloroplast genome size with LSC, SSC, and IR sizes. **(A)** Chloroplast genome annotation of the genus *Hordeum*, with accession number 95–3 as a representative. **(B-D)** Scatter plots with linear regression lines (red) and 95% confidence intervals (shaded) illustrate the relationships of Poaceae chloroplast genome size with LSC, SSC, and IR sizes. Pearson correlation coefficients (r) and corresponding p-values are shown in each panel. **(E)** Statistical analysis of the coefficients of variation for LSC, SSC, and IR sizes in Poaceae.

We assembled and annotated 43 chloroplast genomes from 12 *Hordeum* species (**Figure 1A**; **Supplementary Table 2**). All chloroplast genomes exhibited a quadripartite structure separated by IRs, with sizes ranging from 136,461 to 137,149 bp. The LSC length ranged from 80,596 to 81,221 bp, SSC from 12,701 to 12,799 bp, and IR from 43,144 to 43,216 bp. GC content ranged from 38.23% to 38.32%. *H. bogdanii* and *H. brevisubulatum* possessed slightly larger chloroplast genomes, while *H. bulbosum* and *H. vulgare* had smaller ones. All 12 *Hordeum* chloroplast genomes contained 83 protein–coding genes (76 unique protein–coding genes), 47–48 tRNA genes (three *H. pusillum* accessions had 46 tRNA genes, lacking one *trnT-GGU*), and 8–9 rRNA genes.

For comparison, we re–annotated 18 chloroplast genomes of other Poaceae species (**Supplementary Table 2**). These chloroplast genomes also exhibited the typical quadripartite structure, with genome sizes ranging from 134,297 to 140,754 bp, LSC from 79,502 to 83,733 bp, SSC from 12,335 to 12,830 bp, and IR from 41,598 to 45,554 bp. GC content ranged from 36.65% to 38.99%. All Poaceae chloroplast genomes contained 82-84 protein–coding genes (76 unique protein–coding genes), 46–47 tRNA genes, and 8 rRNA genes, indicating high conservation of coding gene content across Poaceae.

Correlation analysis between chloroplast genome size and the lengths of LSC, SSC, and IR in the 61 Poaceae chloroplast genomes revealed that the IR region had the highest coefficient of variation (1.46%; **Figures 1B–E**). LSC and IR showed the strongest positive correlations with genome size (Pearson r = 0.853 and 0.857, respectively), whereas the correlation for SSC was nearly zero (r = -0.099), indicating that chloroplast genome size in the studied grasses is jointly determined by LSC and IR.

### 3.2 Chloroplast genome phylogeny

**Figure 2:**
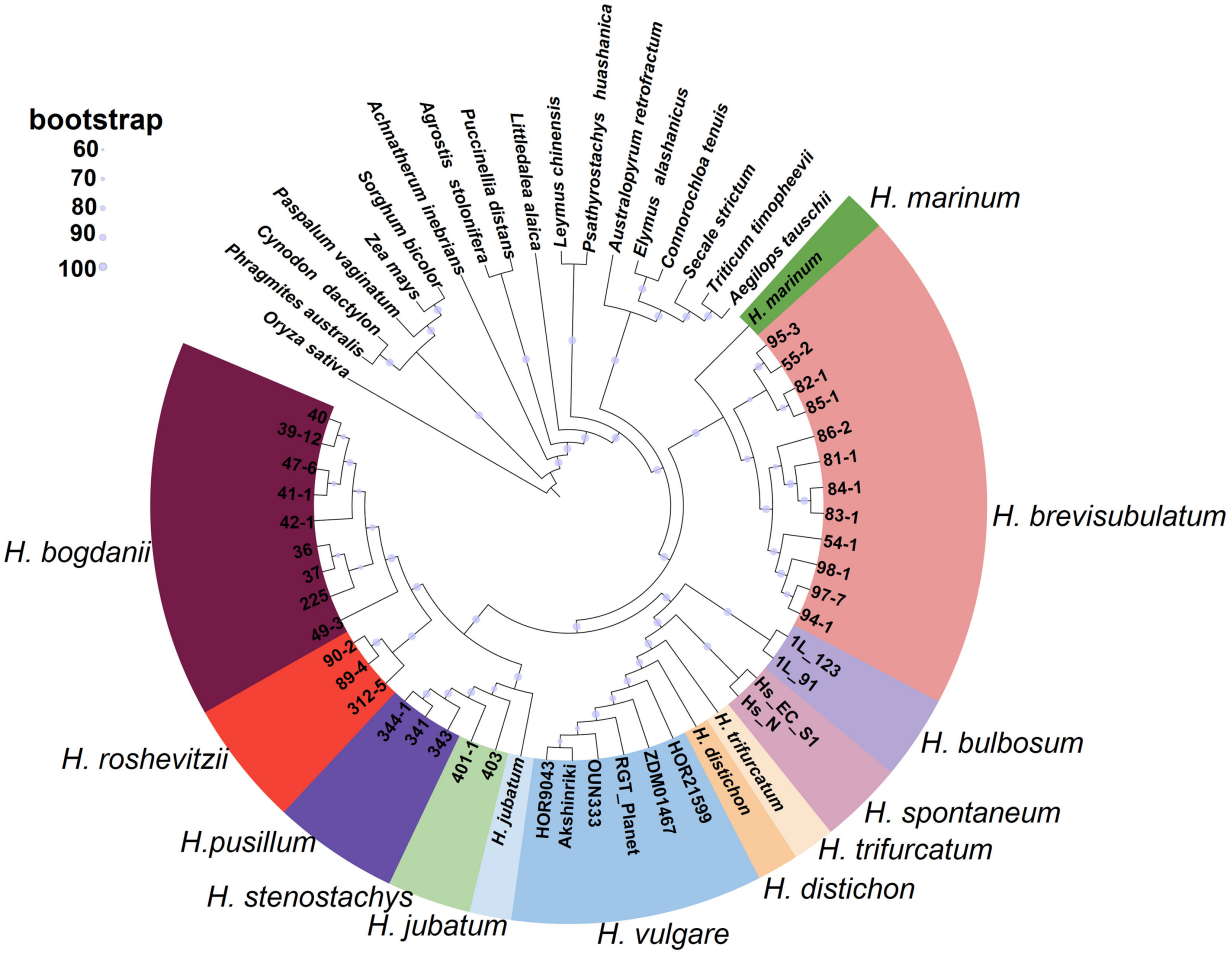
ML phylogenetic tree constructed using whole chloroplast genomes of *Hordeum* species and other Poaceae species. The dot sizes on the nodes are proportional to the bootstrap support values.

Using *Oryza sativa* as an outgroup, we constructed an ML tree based on the 43 *Hordeum* chloroplast genomes and 18 other Poaceae chloroplast genomes (**Figure 2**). Within *Hordeum*, three major clades were recovered: Clade I included *H. bogdanii*, *H. roshevitzii*, *H. pusillum*, *H. stenostachys*, and *H. jubatum*; Clade II comprised *H. vulgare*, *H. distichon*, *H. trifurcatum*, *H. spontaneum*, and *H. bulbosum*; and Clade III consisted of *H. brevisubulatum* and *H. marinum*. Within Triticeae, the Triticinae species (*Triticum timopheevii*, *Aegilops tauschii*, *Secale strictum*) initially formed a clade with the Hordeinae species *Elymus alashanicus*, *Connorochloa tenuis*, and *Australopyrum retrofractum*. This combined clade subsequently clustered with *Hordeum* species. Outside this group were other Hordeinae (*Leymus chinensis*, *Psathyrostachys huashanica*) and Littledaleae (*Littledalea alaica*).

### 3.3 Chloroplast genome rearrangements and inversions

Synteny analysis showed that *Hordeum* species shared many syntenic blocks (**Supplementary Figure 1**). *H. marinum* exhibited putative small inversions (105,000–115,000 bp). Two *H. bulbosum* accessions (1L_91 and 1L_123) showed putative large rearrangements (100,000–115,000 bp) compared with other *Hordeum* species. For validation of the above rearrangements, we re-aligned the Illumina sequencing reads of *H. marinum* and the HiFi sequencing reads of *H. bulbosum* to their respective chloroplast genomes, and visualized the read distribution at the rearrangement regions (**Supplementary Figure 3**), there is sufficient distribution of reads in above rearrangement area, but further long-read evidence is still needed for *H. marinum*. Among other Poaceae species, *Connorochloa tenuis* and *Elymus alashanicus* shared more syntenic blocks with *Hordeum*, while the remaining species shared fewer.

### 3.4 IR expansion/contraction

**Figure 3:**
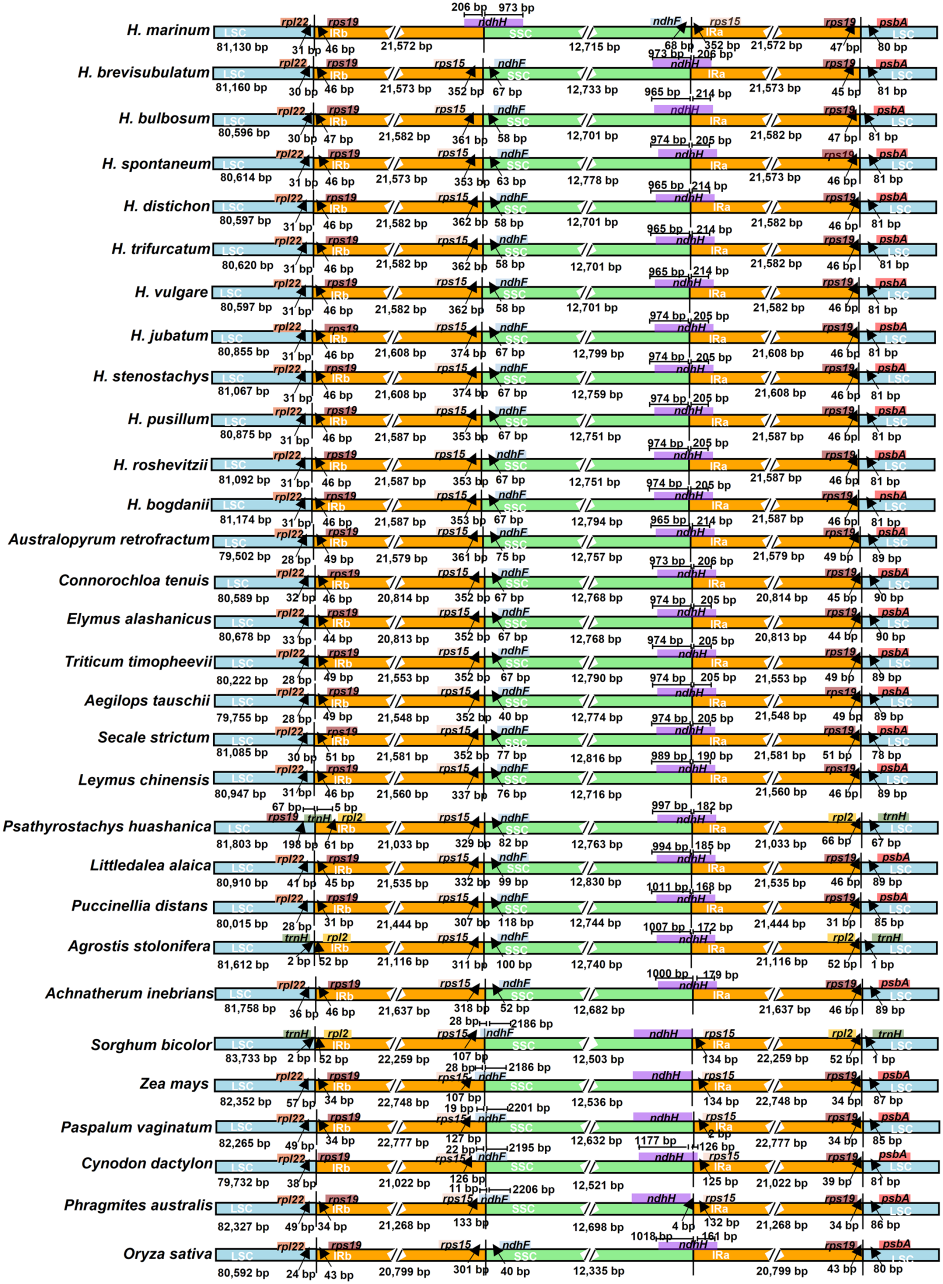
IRscope analysis of 12 *Hordeum* chloroplast genomes and 18 Poaceae chloroplast genomes.

We examined the boundaries between IR and single-copy regions in *Hordeum* and other Poaceae species using one representative per *Hordeum* species (**Figure 3**). Within *Hordeum*, the boundary structure was highly conserved: *rpl22* (30–31 bp from the boundary) and *rps19* (46–47 bp) were located at the LSC/IRb boundary; *psbA* (80–81 bp) and *rps19* (45–47 bp) at the LSC/IRa boundary. In all *Hordeum* species except *H. marinum*, *ndhF* (58–67 bp) and *rps15* (352–374 bp) were located at the SSC/IRb boundary, and *ndhH* spanned the SSC/IRa boundary (965–974 bp into SSC, 205–214 bp into IRa).

Based on IR boundary differences, the 18 Poaceae chloroplast genomes could be divided into three types. Type I (including *Australopyrum retrofractum*, *Connorochloa tenuis*, *Elymus alashanicus*, *Triticum timopheevii*, *Aegilops tauschii*, *Secale strictum*, *Leymus chinensis*, *Littledalea alaica*, *Puccinellia distans*, *Achnatherum inebrians*, and *Oryza sativa*) showed boundary structures and gene features similar to those of *Hordeum*. Type II (including *Zea mays*, *Paspalum vaginatum*, *Cynodon dactylon*, and *Phragmites australis*) had *ndhF* crossing the SSC/IRb boundary, while *ndhH* remained within SSC (except in *Cynodon dactylon*, where *ndhH* still spanned SSC/IRa). Type III (including *Psathyrostachys huashanica*, *Agrostis stolonifera*, and *Sorghum bicolor*) was characterized by *trnH* and *rpl2* located at the LSC/IRb and LSC/IRa boundaries; *ndhH* crossed the SSC/IRa boundary in *Psathyrostachys huashanica* and *Agrostis stolonifera*, but remained entirely within SSC in *Sorghum bicolor*.

### 3.5 Repeat sequence analysis

**Figure 4:**
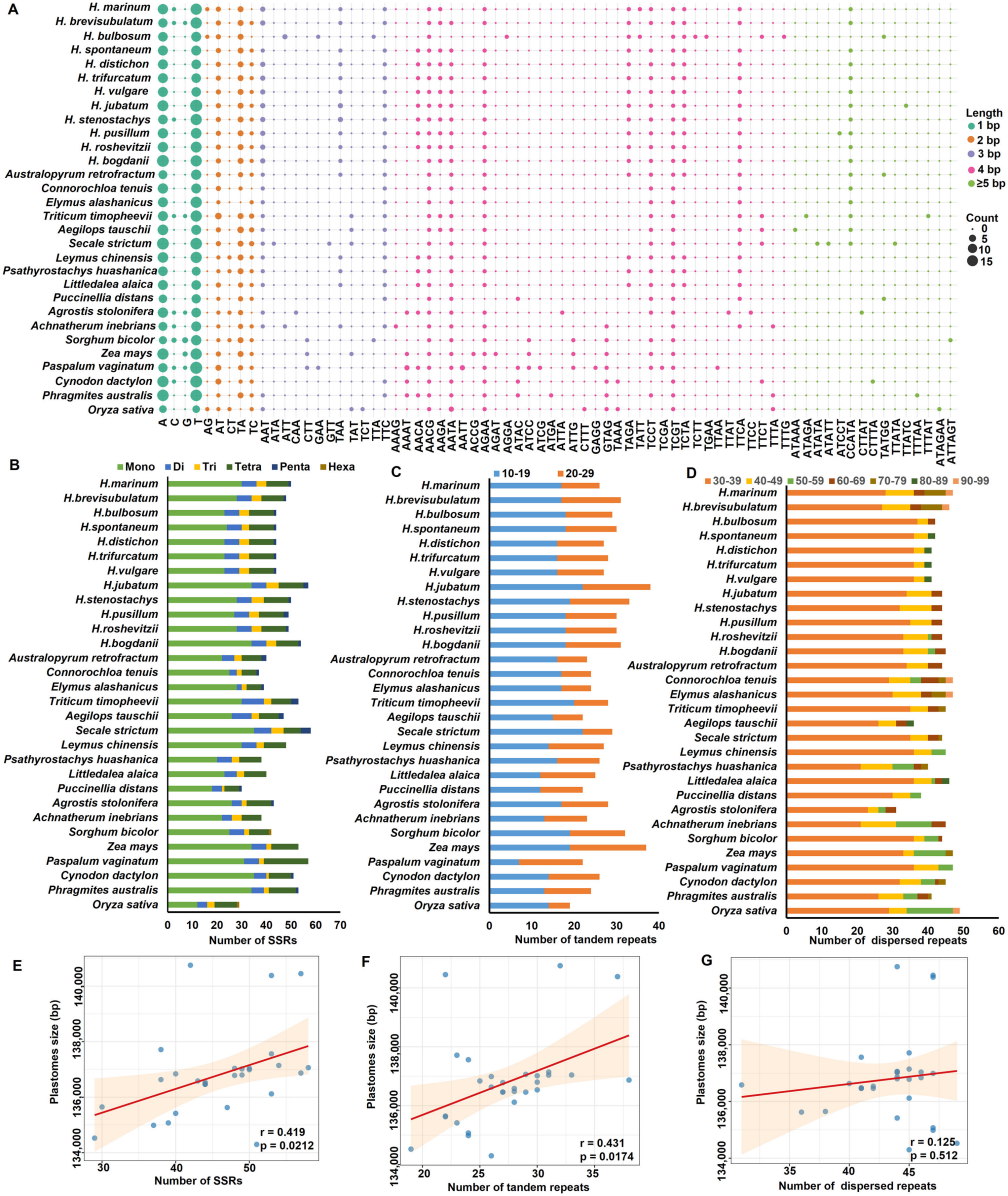
Repeat sequence analysis of 12 *Hordeum* chloroplast genomes and 18 Poaceae chloroplast genomes. **(A)** Statistical analysis of the counts of different SSR types in chloroplast genomes. **(B-D)** Statistical analysis of the numbers of SSRs, tandem repeats, and dispersed repeats classified by length in different chloroplast genomes. **(E-G)** Correlation analysis between chloroplast genome size and the numbers of SSRs, tandem repeats, and dispersed repeats. Scatter plots were shown with linear regression lines (red) and 95% confidence intervals (shaded). Pearson correlation coefficients (r) and corresponding p-values are shown in each panel.

A total of 557 SSRs were identified in the 12 *Hordeum* chloroplast genomes (**Figures 4A, B**). *H. jubatum* contained the highest number (57 SSRs), while *H. bulbosum*, *H. spontaneum*, *H. distichon*, *H. trifurcatum*, and *H. vulgare* had the lowest (44 SSRs each). Across the 18 other Poaceae chloroplast genomes, 796 SSRs were identified (**Figures 4A, B**), with *Secale strictum* having the most (58 SSRs) and *Oryza sativa* the fewest (29 SSRs). A/T mononucleotide repeats were the most common SSRs (average 56% in *Hordeum*, 59% in other Poaceae). Hexanucleotide SSRs (ATAGAA, ATTAGT) were found only in *Oryza sativa* and *Sorghum bicolor*. All *Hordeum* species examined possessed pentanucleotide SSRs (*H. jubatum*: CCATA and TTATC; *H. pusillum*: ATCCT, CCATA; the others: CCATA or TATGG). *Secale strictum* had the highest number of pentanucleotide SSRs (ATATA, ATATT, CCATA, TTATA), whereas *Achnatherum inebrians*, *Leymus chinensis*, *Littledalea alaica*, *Oryza sativa*, *Paspalum vaginatum*, *Psathyrostachys huashanica*, *Sorghum bicolor*, and *Zea mays* lacked pentanucleotide SSRs entirely.

Most tandem repeats were shorter than 20 bp (**Figure 4C**). The 12 *Hordeum* chloroplast genomes contained 360 tandem repeats (213 of 10–19 bp and 147 of 20–29 bp); *H. jubatum* had the highest numbers (22 of 10–19 bp and 16 of 20–29 bp). The 18 other Poaceae chloroplast genomes contained 461 tandem repeats (277 of 10–19 bp and 184 of 20–29 bp); *Secale strictum* had the most 10–19 bp repeats (22), and *Zea mays* had the most 20–29 bp repeats (18). Dispersed repeats were predominantly 30–39 bp in length (**Figure 4D**). The 12 *Hordeum* chloroplast genomes contained 521 dispersed repeats, and the pattern of dispersed repeat types in each *Hordeum* species was related to its phylogenetic clustering. The 18 Poaceae chloroplast genomes contained 781 dispersed repeats, with large variation among genera.

Correlation analysis between chloroplast genome size and the numbers of SSRs, tandem repeats, and dispersed repeats revealed that chloroplast genome size was positively correlated with the number of tandem repeats (r = 0.431) and SSRs (r = 0.419), but not with dispersed repeats (**Figures 4E–G**).

### 3.6 Codon usage

**Figure 5:**
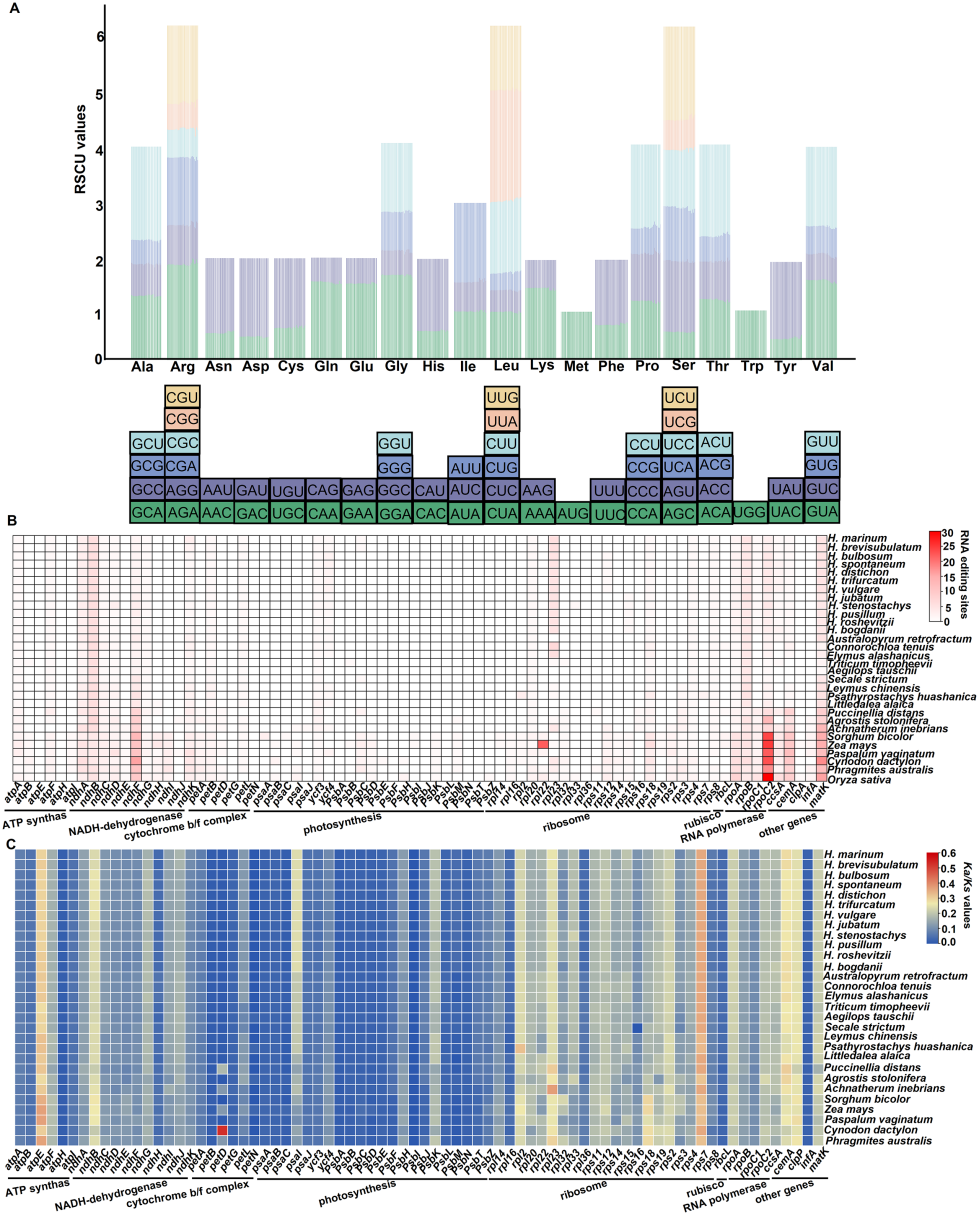
Analysis of codon usage, RNA editing sites, and selective pressure in chloroplast genomes of *Hordeum* and other Poaceae species. **(A)** RSCU values across 30 chloroplast genomes. The order of species in the bar chart is consistent with that in panels **(B, C)** RNA editing sites identified in 66 protein–coding genes. The scale bar represents the number of identified RNA editing sites. **(C)** *Ka/Ks* ratios of 76 protein–coding genes. The scale bar represents the *Ka/Ks* values.

RSCU values were calculated to evaluate codon usage frequency. A total of 61 codons encoding 20 amino acids were identified, with leucine (Leu) being the most abundant and cysteine (Cys) the least abundant. Codon usage trends were similar across the 12 *Hordeum* chloroplast genomes and the 18 other Poaceae chloroplast genomes (**Figure 5A**), with clear bias among synonymous codons. We identified 29 codons with RSCU > 1, indicating they are used more frequently than others. The most frequently used codon was UUA (encoding Leu), with RSCU > 2 in all *Hordeum* species, but in only some other Poaceae species. The least frequently used codon was CUG (also encoding Leu).

### 3.7 RNA editing sites

A total of 66 protein–coding genes were found to contain RNA editing sites across the 12 *Hordeum* and 18 other Poaceae chloroplast genomes (**Figure 5B**). In the 12 *Hordeum* chloroplast genomes, *ndhB* had the highest number of editing sites (6), followed by *rpl23* and *matK*. Among the other Poaceae species, *Oryza sativa* had the highest number of editing sites (30), and the largest interspecific variation was observed for *rpoC2*. Additionally, *matK*, *cemA*, *rpoA*, and *ndhF* showed lower editing site numbers in *Hordeum* but higher numbers in other Poaceae species such as *Achnatherum inebrians*, *Sorghum bicolor*, *Zea mays*, *Paspalum vaginatum*, *Cynodon dactylon*, *Phragmites australis*, and *Oryza sativa*.

### 3.8 Positive/purifying selection analysis

Using *Oryza sativa* as a reference, we calculated the nonsynonymous (*Ka*) and synonymous (*Ks*) substitution rates for the 76 protein-coding genes in Poaceae (**Figure 5C**). Overall, all *Ka/Ks* ratios were far below 1, demonstrating that all genes have experienced varying degrees of purifying selection. *rps7* exhibited the highest *Ka/Ks* ratio, indicating the weakest purifying selection. Ribosomal subunit genes showed the largest interspecific variation in *Ka/Ks*, suggesting that these genes have been subject to differential purifying selection during evolution of chloroplast genomes in Poaceae.

### 3.9 Nucleotide polymorphism and genetic variation

We compared chloroplast genome sequence divergence and identity across the 12 *Hordeum* species using *H. vulgare* (NC_008590) as a reference. Variation was concentrated in the LSC region (**Supplementary Figure 2**), and the main variable genes included *psbZ*, *psbM*, *petN*, *trnfM-CAU*, *trnG-UCC*, *trnE-UUC*, *trnD-GUC*, *trnC-GCA*, *trnM-CAU*, *trnY-GUA*, *trnT-GGU*, etc.

**Figure 6:**
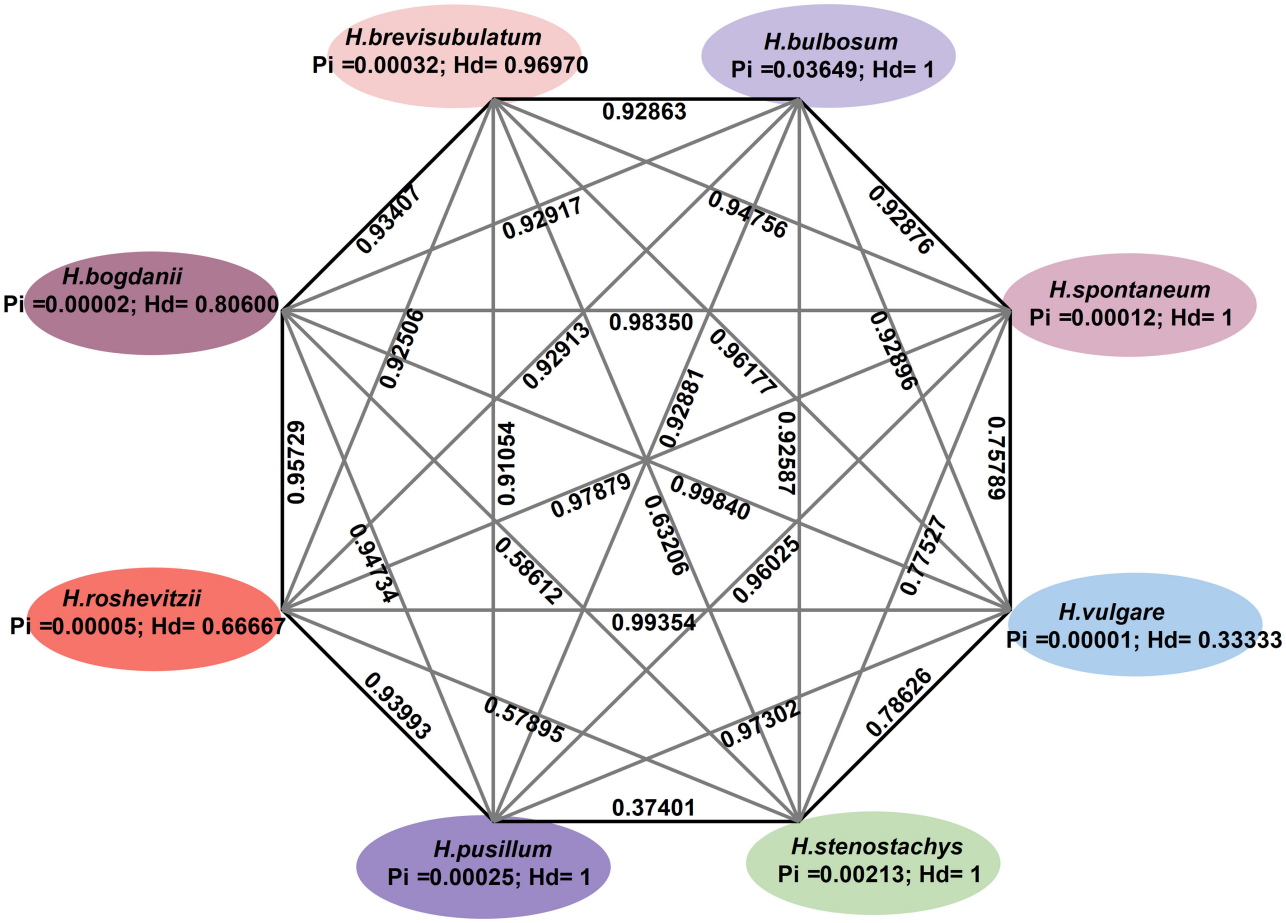
Nucleotide polymorphism (Pi), haplotype diversity (Hd), and pairwise genetic differentiation (Fst) among chloroplast genomes of eight *Hordeum* species.

Nucleotide polymorphism analysis was performed on eight *Hordeum* species (12 accessions of *H. brevisubulatum*, two accessions of *H. bulbosum*, two accessions of *H. spontaneum*, six accessions of *H. vulgare*, two accessions of *H. stenostachys*, three accessions of *H. pusillum*, three accessions of *H. roshevitzii*, and nine accessions of *H. bogdanii*), and Pi and Hd were calculated for each species. The Pi values of the eight *Hordeum* species ranged from 0.00001 to 0.03649, and Hd values from 0.33333 to 1 (**Figure 6**). *H. bulbosum* exhibited the highest intraspecific genetic variation (Pi = 0.03649, Hd = 1), followed by *H. stenostachys*, *H. brevisubulatum*, *H. pusillum*, *H. spontaneum*, *H. roshevitzii*, and *H. bogdanii*. *H. vulgare* showed the lowest intraspecific diversity (Pi = 0.00001, Hd = 0.33333). Pairwise Fst values indicated the greatest interspecific differentiation between *H. bogdanii* and *H. vulgare* (Fst = 0.99840), followed by *H. roshevitzii* vs. *H. vulgare* (Fst = 0.99354), while *H. stenostachys* exhibited relatively low levels of differentiation with *H. pusillum*, *H. roshevitzii*, *H. bogdanii*, and *H. brevisubulatum*.

## 4 Discussion

Through systematic pan-chloroplast analysis of 43 genomes from 12 *Hordeum* species and 18 other Poaceae species, this study reveals key features of chloroplast genome structure, evolution, and genetic diversity. Our results provide important data and theoretical support for studies on phylogeny, genome evolution, and adaptation in *Hordeum*.

### 4.1 Conservation and lineage-specific variation of chloroplast genome structure

All *Hordeum* and Poaceae chloroplast genomes examined exhibited the typical quadripartite structure, with overall conservation of genome size, GC content, and gene number, consistent with previous observations in Poaceae (Skuza et al., 2025; Zhang et al., 2025). Within *Hordeum*, however, *H. bogdanii* and *H. brevisubulatum* had slightly larger chloroplast genomes, while *H. bulbosum* and *H. vulgare* had smaller ones, and this difference mainly originated from length variation in the LSC and IR regions. Correlation analysis further confirmed that LSC and IR lengths were strongly positively correlated with genome size (Pearson r > 0.85), whereas SSC contributed negligibly, indicating that IR expansion/contraction and LSC insertions/deletions are the dominant drivers of chloroplast genome size variation in Poaceae. The higher coefficient of variation of IR (1.46%) compared with LSC and SSC also supports the greater dynamism of IR during evolution.

In IR boundary analysis, *Hordeum* species displayed a highly consistent gene distribution pattern with stable positions of *rpl22*, *rps19*, *rps15, ndhF*, *ndhH*, *psbA*, etc., contrasting with a previous study on six *Hordeum* species that reported marked annual-perennial differences (Yuan et al., 2023). The discrepancy may stem from different taxon sampling, as we observed only *H. marinum* as an exception. Among the 18 Poaceae chloroplast genomes, three major boundary types were identified. Type I is relatively consistent with previous results (Skuza et al., 2025), whereas Types II and III appear to represent configurations that, to our knowledge, have not been previously documented in Poaceae. These differences imply that repeated IR expansion and contraction occurred during Poaceae evolution, serving as an important source of lineage−specific structural variation, and underscore the evolutionary lability of IR boundaries across the Poaceae family. Additionally, synteny analysis revealed putative small inversions in *H. marinum* and putative large rearrangements in two *H. bulbosum* accessions. Such structural rearrangements are relatively rare in chloroplast genomes and warrant further confirmation by long–read sequencing. We used long reads to validate the rearrangement region in *H. bulbosum*, but did not perform the same validation in *H. marinum*, owing to the lack of available data.

### 4.2 Distribution of repeats and their contribution to genome evolution

Our repeat analysis generally corroborates previous findings in *Hordeum* (Yuan et al., 2023) and *Secale strictum* (Skuza et al., 2025): A/T mononucleotide repeats were predominant (averaging 56% in *Hordeum* and 59% in other Poaceae), directly reflecting the AT−rich nature of chloroplast genomes. Repeat analysis further showed that the numbers of SSRs and tandem repeats were positively correlated with chloroplast genome size (r = 0.419 and 0.431), confirming that repeat expansion is another important driver of genome size variation. Pentanucleotide repeat motifs were present in all *Hordeum* species examined but absent in several other Poaceae species examined such as *Oryza sativa*, *Sorghum bicolor*, and *Zea mays*. This distribution pattern suggests that pentanucleotide repeats were enriched in the sampled *Hordeum* chloroplast genomes and may be useful candidate markers pending validation across broader taxonomic and population sampling. The types of dispersed repeats in each *Hordeum* species were related to its phylogenetic clustering, implying gain or loss of dispersed repeats during lineage divergence.

### 4.3 Phylogenetic relationships reconstructed from chloroplast genomes

Classic phylogenetic studies in *Hordeum* have repeatedly reported conflicts between trees constructed based on chloroplast and nuclear genes (Jakob and Blattner, 2006). In this study, the ML tree based on chloroplast genomes resolved three major clades within *Hordeum*. This topology is largely consistent with previous studies also constructed based on *Hordeum* chloroplast genomes (Yuan et al., 2023). However, the phylogenetic tree based on chloroplast genomes does not adequately resolve the evolutionary relationships among the different genome types (H, I, Xa, Xu) of *Hordeum* that were previously established based on nuclear genes (Blattner, 2004). A noteworthy observation is the grouping of *H. pusillum* (North American distribution) with the Eurasian *H. bogdanii* and *H. roshevitzii*, suggesting that the chloroplast of *H. pusillum* may have originated from hybridization and introgression with an ancestral Eurasian lineage. The close clustering of *H. brevisubulatum* and *H. marinum* also deserves attention because the former is perennial and the latter annual, yet both are strongly salt–tolerant; their high chloroplast genome similarity might reflect recent divergence or convergent selection under saline conditions.

### 4.4 Coordinated evolution of codon usage, RNA editing, and selective pressure

Codon usage bias analysis revealed that all *Hordeum* and Poaceae chloroplast genomes exhibit pronounced synonymous codon usage bias, with the leucine-encoding UUA having the highest RSCU (> 2 in all *Hordeum* species) and CUG the lowest. This bias is related to the high AT content of chloroplast genomes and selection pressure for translational efficiency. RNA editing sites were predicted in 66 protein–coding genes, with *ndhB* containing the highest number (six), followed by *rpl23* and *matK*. Notably, the numbers of editing sites in genes such as *rpoC2*, *matK*, and *cemA* varied considerably among Poaceae species, whereas these numbers remained relatively stable within *Hordeum*.

All examined protein–coding genes had *Ka/Ks* ratios far below 1, confirming that purifying selection appears to dominate chloroplast genome evolution. *rps7* had the highest *Ka/Ks*, indicating the weakest evolutionary constraint, possibly related to its accessory role in ribosome assembly. In contrast, ribosomal subunit genes showed the largest interspecific variation in *Ka/Ks*, suggesting that these genes have experienced differential purifying selection.

### 4.5 Hypervariable regions, nucleotide polymorphism and genetic differentiation

Variation was unevenly distributed across the chloroplast genome, being concentrated in several intergenic regions and coding regions within the LSC (e.g., *psbZ*, *trnG-UCC*). These hypervariable regions provide direct targets for developing *Hordeum*-specific DNA barcodes, intraspecific identification markers, and population genetic studies.

Analysis based on multiple individuals per species showed that *H. bulbosum* harbored the highest nucleotide diversity and haplotype diversity, indicating that this species retains abundant genetic variation. In contrast, the six sampled *H. vulgare* accessions, all with domestication backgrounds, showed very low chloroplast genome diversity in this dataset; broader sampling of wild barley, landraces, and cultivars is needed for further verify domestication-related chloroplast genome diversity loss. Interspecific differentiation (Fst) revealed the largest divergence between *H. bogdanii* and *H. vulgare*, whereas the smallest divergence was observed between *H. stenostachys* and *H. pusillum*, suggesting that *H. stenostachys* and *H. pusillum* may share a recent common ancestor or have experienced frequent historical gene flow, possibly due to incomplete lineage sorting or ongoing hybridization.

## 5 Conclusion

In the current study, the comprehensive pan-chloroplast analysis of the genus *Hordeum* was performed to describe structural, evolutionary, and diversity features. The *Hordeum* chloroplast genome has a typical quadripartite structure, and its size variation is primarily driven by the LSC and IR regions. The IR boundaries are highly conserved within the genus, whereas the putative rearrangements detected in *H. marinum* and *H. bulbosum* suggest the potential for structural variation. Repetitive sequences are important drivers of genome size and structural variation: the numbers of SSRs and tandem repeats are positively correlated with chloroplast genome size. Pentanucleotide repeats are present in all *Hordeum* species examined and represent a candidate *Hordeum*-enriched marker pending broader validation. The chloroplast genome tree recovered three major chloroplast lineages within the sampled *Hordeum* accessions. Strong purifying selection appears to dominate chloroplast genome evolution, all examined protein–coding genes show *Ka/Ks* values below 1, but ribosomal subunit genes exhibit differential selective constraints among species. Genetic diversity varies markedly, and hypervariable regions are identified: *H. bulbosum* shows the highest diversity, whereas *H. vulgare* shows the lowest. The hypervariable regions identified provide important resources for future population genetics and molecular marker development. These findings not only deepen our understanding of chloroplast genome evolution in *Hordeum* and Poaceae, but also lay a solid molecular foundation for phylogenetic reconstruction, germplasm identification, and adaptive evolution studies in the genus. Future integration of nuclear genome data for nuclear-cytoplasmic joint analysis will be a key step toward revealing the complete evolutionary picture of *Hordeum*.

## Data Availability

The original contributions presented in the study are included in the article/**Supplementary Material**. Further inquiries can be directed to the corresponding authors. The newly assembled and annotated chloroplast genomes of the genus Hordeum in this study have been deposited in Sequence Archive of the China National GeneBank Database (CNGBdb, https://db.cngb.org/cnsa/) with accession identifier CNP0009661.
