# Supplementary material for: Pan-chloroplast genomes of Hordeum reveal insights into chloroplast evolution in the Poaceae family
Source: Front Plant Sci. 2026 Jul 20;17:1881945. doi: 10.3389/fpls.2026.1881945 (PMC13429637; doi:10.3389/fpls.2026.1881945)
Supplement: Supplementary file 1 [file DataSheet1.docx]

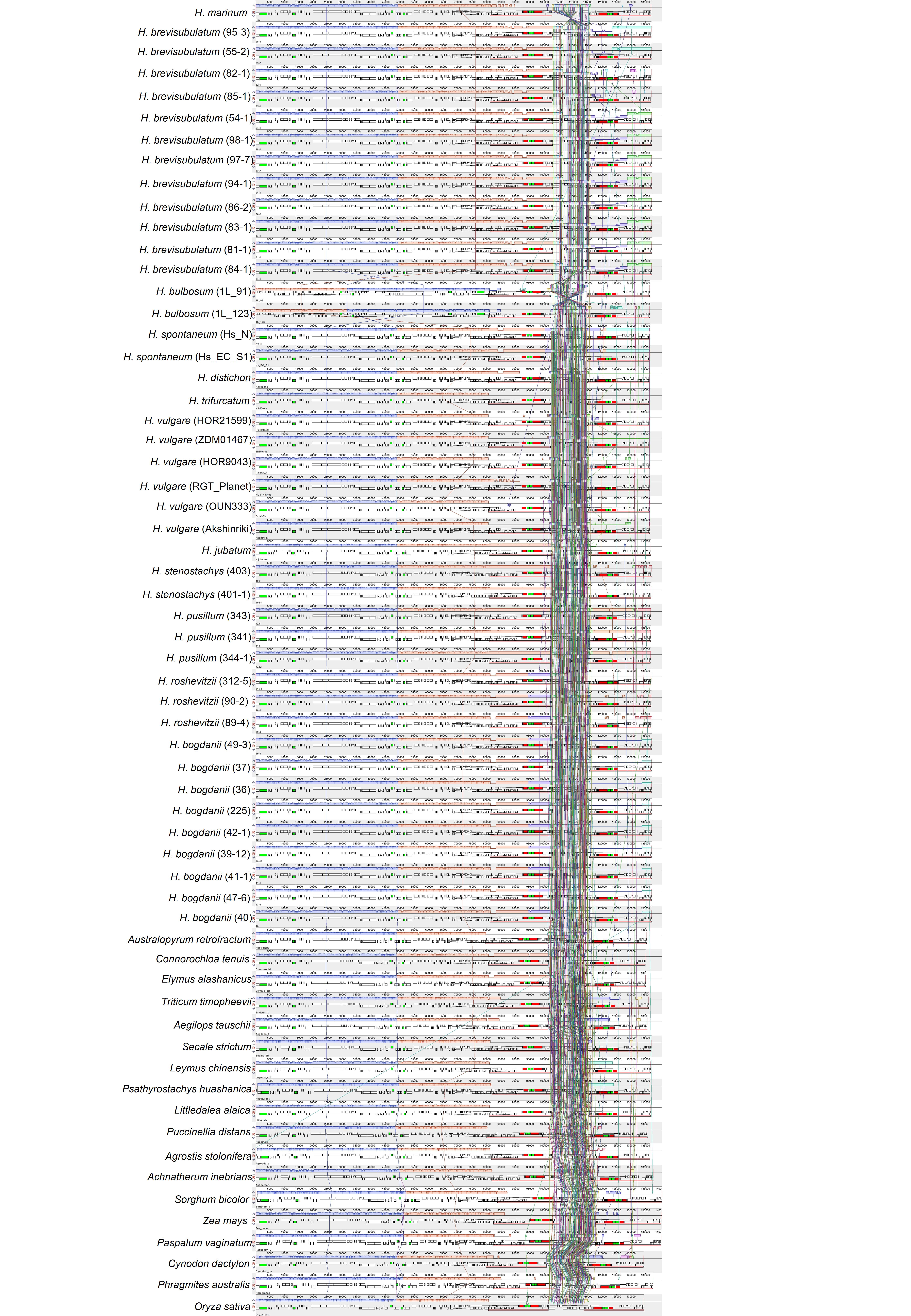


**Supplementary Figure 1.** Synteny analysis of 61 chloroplast genomes.


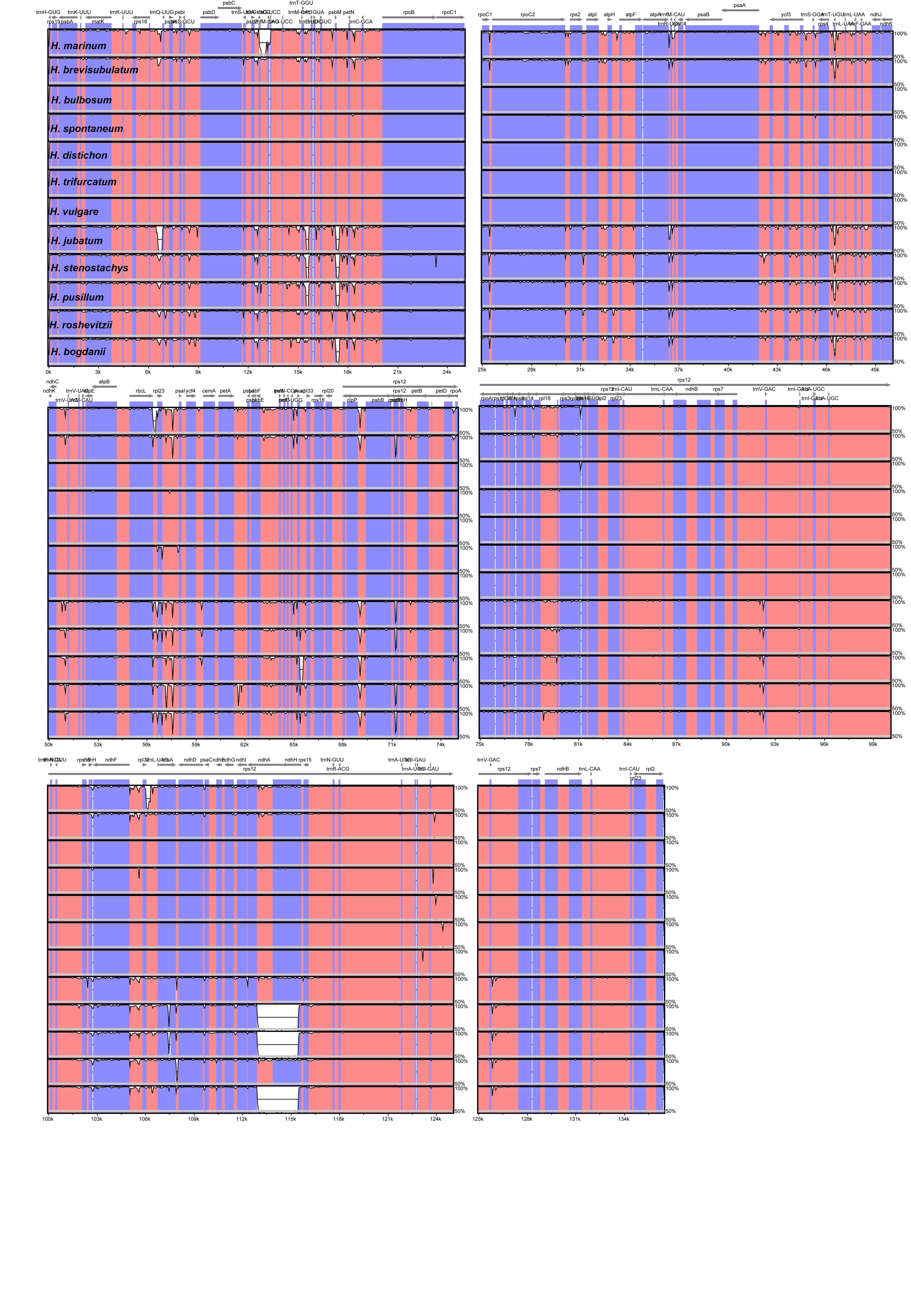


**Supplementary Figure 2.** Chloroplast genome sequence divergence analysis of 12 *Hordeum* species.


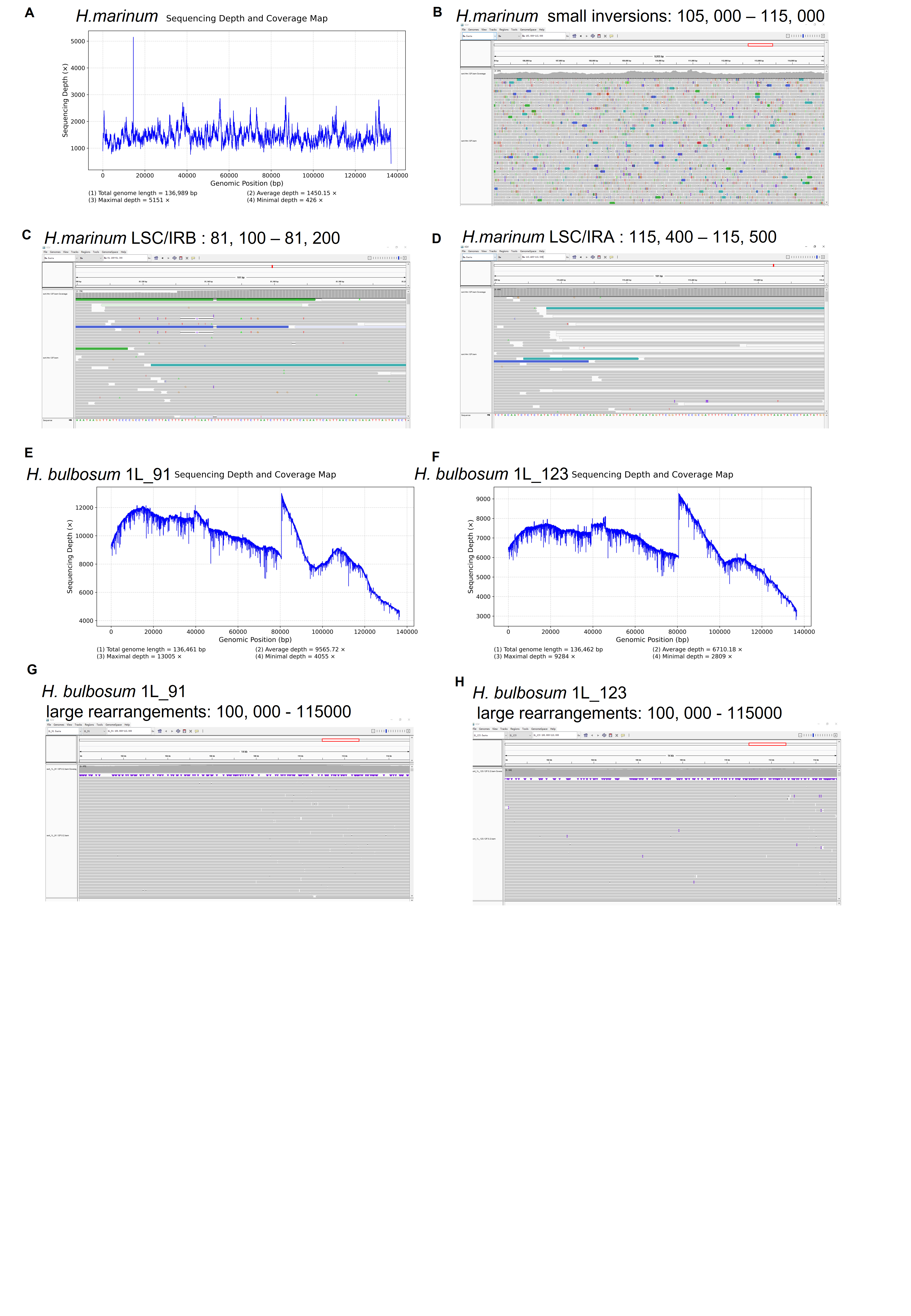


**Supplementary Figure 3.** Validation of the rearrangements reported for *H. marinum* and *H. bulbosum*. (A) Sequence depth and coverage map of *H. marinum*. (B-D) IGVtools shows the read distribution in the small inversions, LSC/IRB, and LSC/IRA regions of *H. marinum*. (E-F) Sequence depth and coverage map of two *H. bulbosum* accessions. (G-H) IGVtools shows the read distribution in the large rearrangements regions of two *H. bulbosum* accessions.
